# Gene expression differences in the olfactory bulb associated with differential social interactions and olfactory deficits in Pax6 heterozygous mice

**DOI:** 10.1242/bio.061647

**Published:** 2025-02-04

**Authors:** Carmen Daems, El-Sayed Baz, Rudi D'Hooge, Zsuzsanna Callaerts-Végh, Patrick Callaerts

**Affiliations:** ^1^ Laboratory of Behavioral and Developmental Genetics, Department of Human Genetics, KU Leuven, 3000 Leuven, Belgium; ^2^ Zoology Department, Faculty of Science, Suez Canal University, 41522 Ismailia, Egypt; ^3^ Laboratory of Biological Psychology, KU Leuven, Leuven, Belgium; ^4^ Mouse behavior core facility mINT, KU Leuven, Leuven, Belgium

**Keywords:** Olfactory bulb, Pax6, Sey mutant, Social behavior, RNA-sequencing

## Abstract

Mutations in the highly conserved *Pax6* transcription factor have been implicated in neurodevelopmental disorders and behavioral abnormalities, yet the mechanistic basis of the latter remain poorly understood. Our study, using behavioral phenotyping, has identified aberrant social interactions, characterized by withdrawal behavior, and olfactory deficits in Pax6 heterozygous mutant mice. The molecular mechanisms underlying the observed phenotypes were characterized by means of RNA-sequencing on isolated olfactory bulbs followed by validation with qRT-PCR. Comparative analysis of olfactory bulb transcriptomes further reveals an imbalance between neuronal excitation and inhibition, synaptic dysfunction, and alterations in epigenetic regulation as possible mechanisms underlying the abnormal social behavior. We observe a considerable overlap with autism-associated genes and suggest that studying Pax6-dependent gene regulatory networks may further our insight into molecular mechanisms implicated in autistic-like behaviors in Pax6 mutations, thereby paving the way for future research in this area.

## Teaser

**Summary:** Pax6 heterozygous mutant mice display aberrant social interactions and olfactory deficits caused by an imbalance between neuronal excitation and inhibition, synaptic dysfunction, and alterations in epigenetic regulation.

## INTRODUCTION

The *PAX6* gene encodes a highly conserved transcription factor with DNA-binding paired and homeodomains (Callaerts et al., 1997). PAX6 has essential roles in the eye, nervous system, olfactory system, and pancreas development (Cvekl and Callaerts, 2017; Nomura et al., 2007; van Heyningen and Williamson, 2002). Heterozygosity for mutations in the human *PAX6* locus causes aniridia, a condition characterized by the complete or partial loss of the iris, resulting in severe visual impairment (Abdolkarimi et al., 2022; Hanson and Van Heyningen, 1995; Ton et al., 1991). Aniridia can appear as an isolated observation or as part of the WAGR (Wilms' tumor, aniridia, genitourinary abnormalities, and mental retardation) syndrome (Hanson and Van Heyningen, 1995; Lee et al., 2008; Ton et al., 1991), which is caused by interstitial deletions in the 11p13 region (Daruich et al., 2023; Yokoi et al., 2016) The complete loss of human PAX6, seen in a case of compound heterozygosity, leads to the absence of eyes, severe CNS abnormalities, and a small deformed nose (Glaser et al., 1994). The important role of PAX6 in CNS development is further reflected in the presence of a range of phenotypes in individuals heterozygous for *PAX6*. These phenotypes not only involve eye abnormalities but also the absence or abnormal structure of the pineal gland, hypoplasia of the anterior commissure, and reduced size of the corpus callosum (Georgala et al., 2011; Sisodiya et al., 2001). Furthermore, mutations in *PAX6* have been implicated in neurodevelopmental disorders such as autism spectrum disorder (ASD) (Kikkawa et al., 2019; Yamamoto et al., 2014), with several patients exhibiting not only eye phenotypes but also behavioral issues like cognitive dysfunction, linguistic impairment, social defects, and motor impairment (Davis et al., 2008; Graziano et al., 2007; Heyman et al., 1999; Maekawa et al., 2009; Malandrini et al., 2001; Ticho et al., 2006). These symptoms overlap significantly with the core symptoms of ASD (Davis et al., 2008; Farmer et al., 2013; Ochi et al., 2022; Xu et al., 2008). However, the mechanistic basis of these behavioral abnormalities remains poorly understood.

Much insight into the role of *PAX6* has been gained from studies in mice. *Small eye* (*Sey*) is a semi-dominant mutation that was shown to be caused by mutations in the mouse *Pax6* gene. Complete loss of *Pax6* in mice is lethal soon after birth and associated with the lack of eyes, nasal cavities, and severe CNS defects (Hogan et al., 1986; Roberts, 1967). *Pax6* heterozygosity in mice results in a range of ocular abnormalities very similar to those observed in humans and affects the development of the CNS and olfactory bulb (Cole et al., 2024; Dellovade et al., 1998; Hill et al., 1991). *Pax6* heterozygosity in rodents (rat and mouse) has been associated with abnormal social behavior, defective ultrasonic vocalization, hyperactivity, cognitive impairments, and altered circadian rhythmicity (Chhabra et al., 2020; Umeda et al., 2010; Yoshizaki et al., 2016). These behavioral abnormalities are reminiscent of some phenotypic components of ASD (Kikkawa et al., 2019; Ochi et al., 2022). However, as in humans, a comprehensive understanding of the mechanisms behind the altered behaviors of *Pax6* heterozygous mice is lacking.

In the current study, we investigated the social interactions of *Pax6* heterozygous mice (*Pax6^Sey/+^*) compared to wild-type (WT) littermates. Moreover, considering the significant role of olfaction in rodent social behavior and the presence of olfactory bulb developmental defects in *Pax6* mutants (Coré et al., 2020; Curto et al., 2014; Dudas et al., 2024; Haba et al., 2009; Imamura and Greer, 2013; Kohwi et al., 2005; Gora et al., 2024), we evaluated the olfactory function of these animals and performed RNA-sequencing on isolated olfactory bulbs to characterize molecular mechanisms underlying the observed phenotypes. Our study revealed social defects and olfactory processing in *Pax6* heterozygous mice and identified genes that were differentially expressed in the olfactory bulb of *Pax6^Sey/+^* mice compared to their WT littermates. Our study adds to the growing body of evidence on how *Pax6* haploinsufficiency affects animal behavior, particularly in the context of neurodevelopmental disorders such as ASD and other conditions characterized by social and sensory processing abnormalities.

## RESULTS

**Fig. 1.**
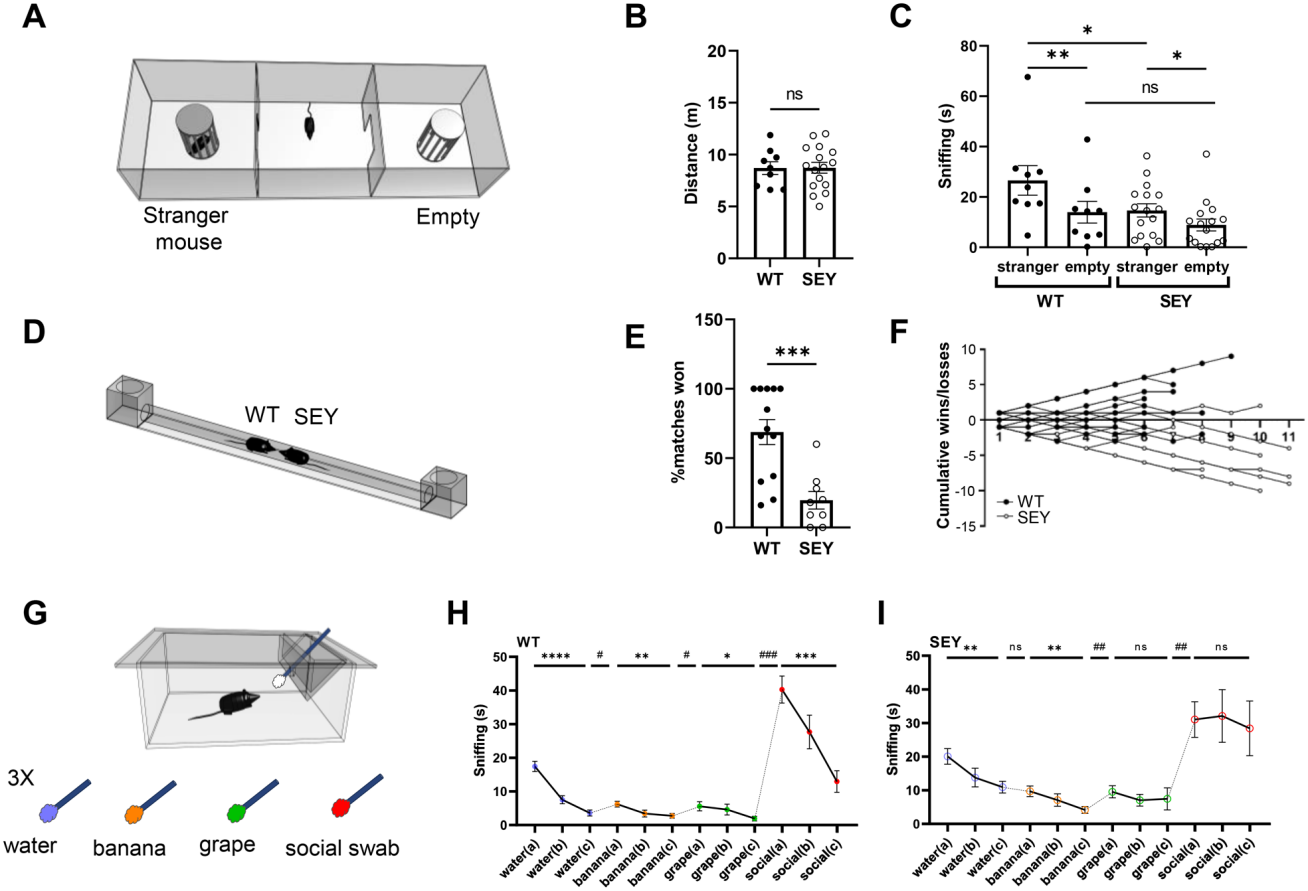
**Social behavior characterization and olfactory habituation/dishabituation assay in Pax6^Sey/+^ (SEY) and WT mice.** (A) Schematic for three-chamber apparatus. During the sociability test, a stranger mouse was placed in a wire cup in one chamber, while an empty cup was placed in the opposite chamber. (B) No difference in the total distance during the sociability test was observed between WT (black circles) and SEY (white circles) animals. (C) WT and SEY mice showed significantly more interest in the stranger mouse in comparison to the empty cage, albeit the difference in SEY mice being only borderline significant (Wilcoxon signed-rank test). WT mice were significantly more interested in the stranger mouse compared to SEY animals (Mann–Whitney test), while there was no difference in interest for the empty side. (D) The tube dominance test assessed social dominance behavior. (E) WT mice were winning significantly more matches in comparison to SEY littermates (unpaired *t*-test). (F) Individual performance of WT and SEY mice in the tube dominance test. (G) Schematic for olfactory habituation/dishabituation assay. Mice were exposed to a series of odors presented on cotton swabs: H_2_O, banana, grape, and social odors. (H,I) Only WT animals showed habituation for the social odor in comparison to SEY mice. Average sniffing time of SEY (I) and WT (H) to water (H_2_O), non-social odors (banana and grape), and social odors. Each odor was presented for 3x 2 min. Presentation of the social odor increased sniffing time for both SEY and WT animals significantly (dishabituation, paired *t*-test for or Wilcoxon matched-pairs signed rank test), whereas only WT animals show a significant habituation response when the social odor is repeatedly presented (across the three trials of the same odor within each genotype: RM one-way ANOVA followed by Sidak's tests, or RM Friedman test ANOVA followed by Dunn's test). Animals used for SPSN: WT=9, SEY=16. Tube dominance test: WT=13; SEY=10. Olfactory assay: WT=11; SEY=16. Data are represented as mean±s.e.m. (dishabituation *P<0.05, **P<0.01, ***P<0.001, ****P<0.0001; habituation ^#^*P*<0.05, ^##^ *P*<0.01, ^###^*P*<0.001, ^####^*P*<0.0001).

The social interactions of *Pax6^Sey/+^* (henceforth referred to as SEY in the results section) and WT mice were evaluated in the social preference and social novelty (SPSN) (Fig. 1A-C) and tube dominance protocol (Fig. 1D-F). Furthermore, the olfactory function was evaluated using the olfactory habituation/dishabituation task (Fig. 1G-I).

During social preference testing, time spent sniffing a stranger mouse was greater than time spent sniffing the empty side in both WT and SEY mice, albeit the difference in SEY mice being only borderline significant (Wilcoxon signed-rank test, *P*=0.0039 for WT and *P*=0.0443 for SEY). WT mice were significantly more interested in the stranger mouse compared to SEY animals (Mann–Whitney test, *P=*0.0478), while there was no difference in interest for the empty side (Mann–Whitney test, *P=*0.2235) (Fig. 1C). The total path length did not differ between genotypes, indicating similar activity in both genotypes during the assay (Fig. 1B). Together, this indicates that SEY mice do not display the same approach behavior towards a conspecific as observed in WT mice.

Social dominance, another aspect of social behavior, was evaluated in the automated tube test (Fig. 1D-F). We observed a clear difference between WT and SEY mice, with WT mice winning significantly more tournaments compared to SEY mice (unpaired *t*-test, *P*=0.0006; Fig. 1E). This submissive phenotype seen in SEY mice corresponds to decreased social approach and increased social avoidance behavior.

Given the importance of odor detection in social behavior and the role of Pax6 in olfactory bulb development, odor detection and discrimination were evaluated in WT and SEY mice using the olfactory habituation/dishabituation task (Fig. 1G-I). During this test, animals are exposed to non-social (water, banana, or grape) and social odors (unfamiliar cage swabs) for three consecutive trials and the time spent sniffing an odor is measured. Habituation to an odor due to repeated exposure is measured as a decrease in sniffing time. An increase in sniffing time when presenting a novel odor is considered dishabituation. We observed that the presentation of H_2_O cotton swabs initially induced some sniffing, but animals quickly lost interest (RM one-way ANOVA, *P*<0.0001 for WT and *P*=0.0052 for SEY). Subsequently, non-social odors elicited very little approach in both genotypes. However, unlike WT, SEY mice showed poor dishabituation between water and the first non-social (banana) odor (water to banana odor: paired *t*-test, *P*=0.03 for WT and *P*=0.5847 for SEY). Both genotypes showed significant habituation for non-social odors [WT: (banana) RM one-way ANOVA, *P*=0.0028 and (grape) RM Friedman test *P*=0.0097; SEY: (banana) RM Friedman test *P*=0.0009 and (grape) RM Friedman test *P*=0.0423. However, post hoc Dunn's test showed no significant difference between grape trials for SEY]. When presented with the social odors, both genotypes showed increased sniffing time, clearly indicating that both genotypes can detect social odors and discriminate between non-social and social odors (grape odor to unfamiliar social odor: Wilkson signed rank tests, *P*=0.001 for WT and *P*=0.0031 for SEY). However, while WT animals exhibited a habituation to social smell, SEY did not (RM one-way ANOVA, *P*<0.0001 and Friedman test ANOVA, *P*=0.7788, respectively). This indicated that SEY mice discriminate between non-social and social odors, but they failed to show habituation when the same social odor cue was repeatedly presented, suggesting a difference in processing social cues.

**Fig. 2.**
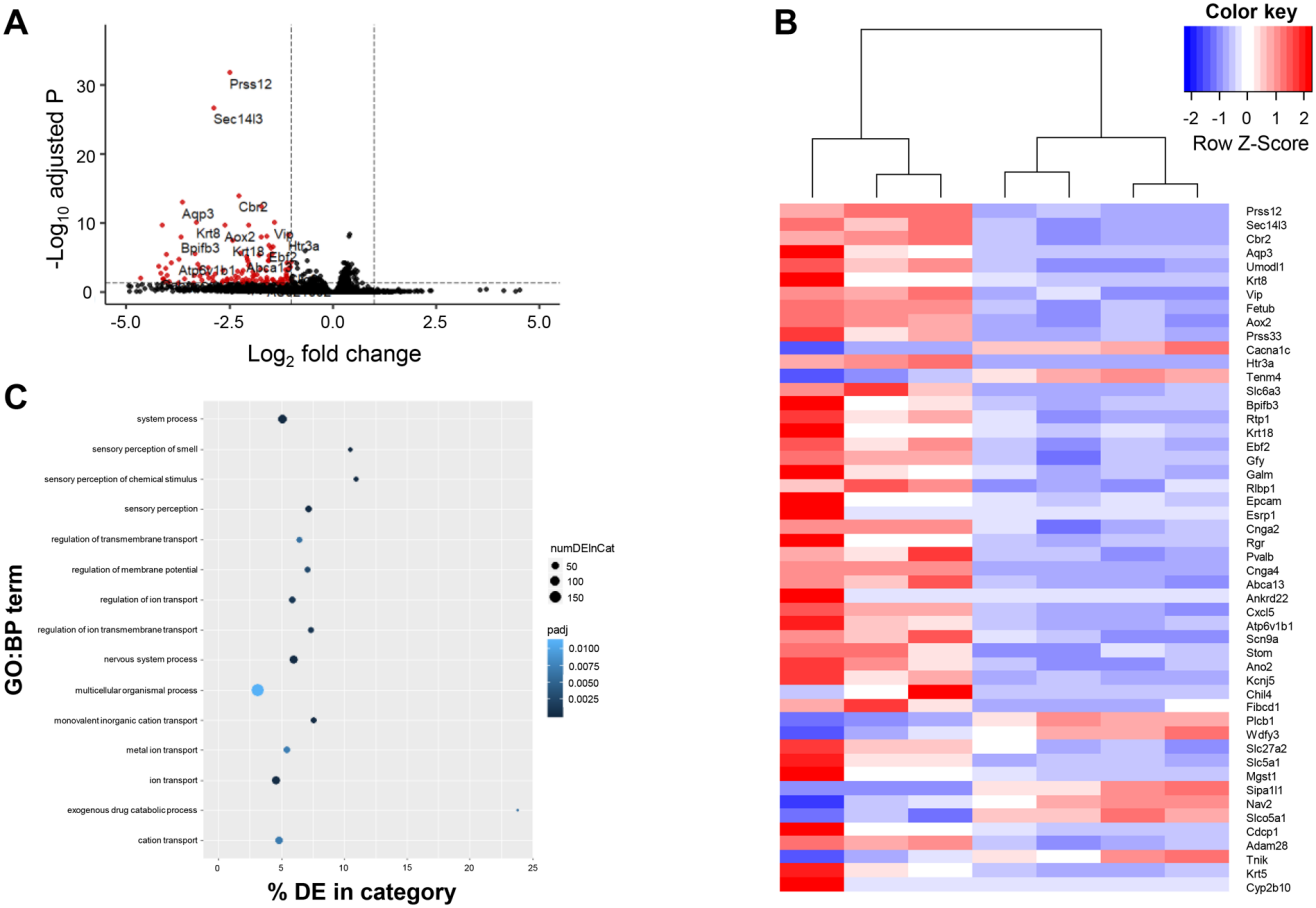
**Overview of RNA-seq results WT and Pax6^Sey/+^ (SEY) mice.** (A) Volcano plot showing the differentially expressed genes (−2<Log_2_ Fold Change>2; FDR<0.05) in red. Names of the top 20 DE genes are indicated. (B) Top 50 differentially expressed genes between WT and SEY mice. (C) Gene Ontology using GOseq shows significant enrichment for terms related to sensory processes, ion channels, and nervous system processes.

To investigate the molecular mechanistic basis underlying the differences in social behavior and the observed differences in social odor processing, RNA-sequencing (RNA-seq) was performed on olfactory bulbs from SEY and WT mice. In total, 317 genes were differentially expressed (FDR<0.05), of which 155 were downregulated and 161 significantly upregulated genes (Fig. 2A,B). Gene ontology mainly indicates enrichment for genes involved in sensory perception of smell, ion transport, and the development of the central nervous system (Fig. 2C). The presence of genes associated with sensory perception among the differentially expressed genes in the olfactory bulb is remarkable, since these genes are known to be expressed in the olfactory epithelium and not in the olfactory bulb (Fig. S1A). We therefore performed qRT-PCR on RNA from carefully isolated olfactory epithelium from SEY and WT mice and were unable to confirm this observation (Fig. S1B,C). We conclude that the presence in the RNA-seq results is most likely due to a small contamination of the olfactory bulb samples with olfactory epithelium.

**Fig. 3.**
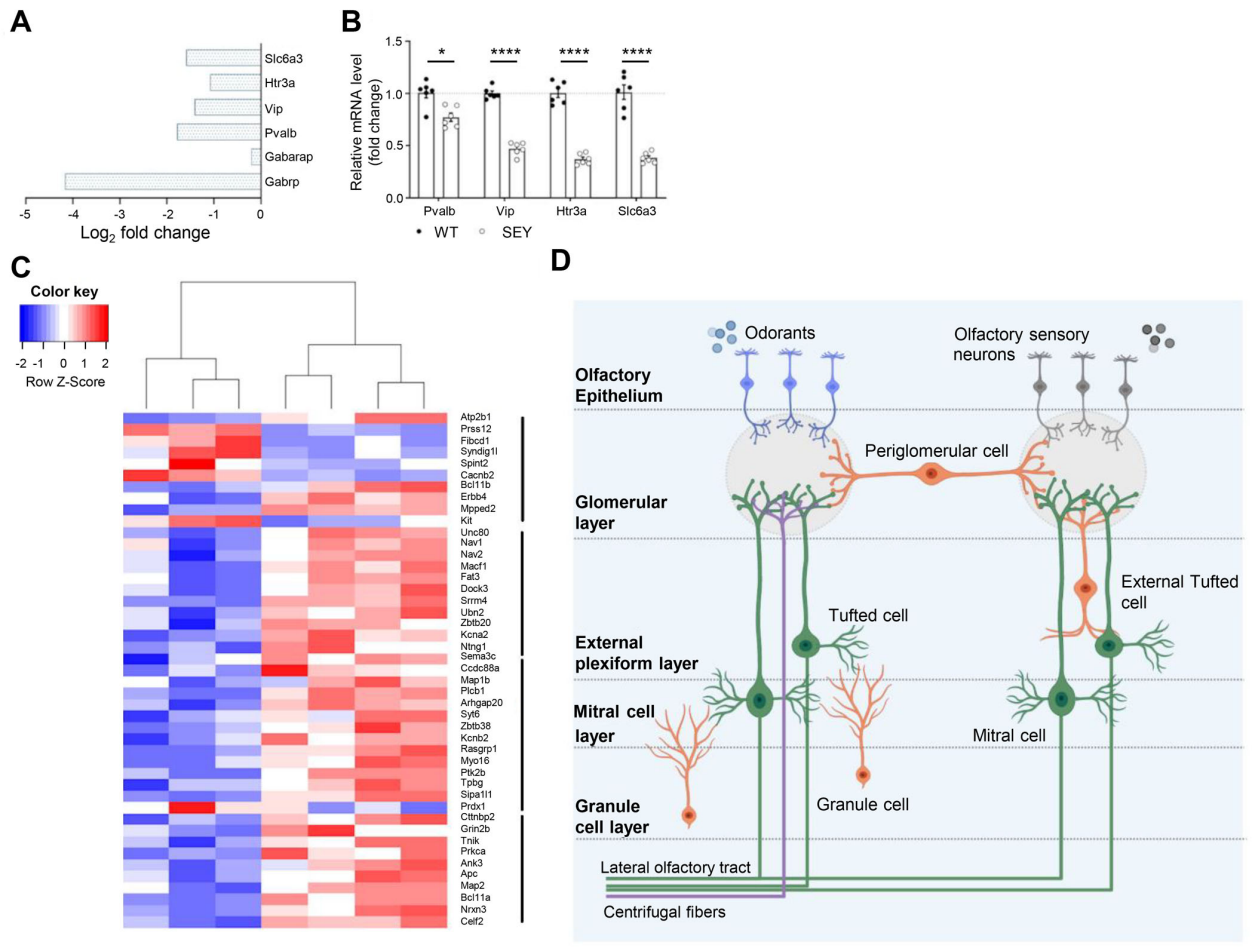
**Significant downregulation of GABAergic-related markers and neuron-specific gene expression.** (A) Markers related to specific subtypes of inhibitory interneurons in the olfactory bulb are significantly downregulated in SEY mice. (B) qRT-PCR validation for markers related to inhibitory interneurons present in the olfactory bulb (*n*=6). (C) Heatmap showing the neuron-specific expression of DE genes between SEY and WT mice. (D) Schematic overview of the olfactory bulb indicating the presence of the major neuronal cell types and different layers. Odorants are detected by olfactory sensory neurons that are present in the olfactory epithelium. This information is transported to glomeruli, where the information is transferred to mitral and tufted (M/T) cells (green). These are the only projection neurons that send the olfactory information to the brain via the lateral olfactory tract. The olfactory output of M/T cells is heavily influenced by GABAergic interneurons (orange). Here, only a subset of GABAergic interneurons relevant to our findings are shown. Parvalbumin-positive (Pvalb) and vasoactive intestinal peptide (VIP)-positive neurons can be found in the external plexiform layer. Dopaminergic (Slc6a3) interneurons are expressed by periglomerular interneurons present in the glomerular layer. Centrifugal fibers (purple) originating from the olfactory cortex can also influence odor information processing. These contain cholinergic, adrenergic, and serotonergic (Htr3a) fibers. This image was created using BioRender.io [Baz, E. (2025) https://BioRender.com/w74r822]. Data are represented as mean±s.e.m., and statistical significance was determined using unpaired *t*-test (B) (*P<0.05, ****P<0.0001).

The olfactory bulb is the first site where the incoming olfactory information is processed before it is projected to the olfactory cortex (Mori et al., 1999). The olfactory signal is transmitted from olfactory sensory neurons to mitral/tufted (M/T) cells in specific glomerular structures within the olfactory bulb (Tan et al., 2010). M/T cells are glutamatergic output neurons of the olfactory bulb, and their activity is heavily influenced by GABAergic interneurons (Huang et al., 2021; Mori et al., 1999; Zechel et al., 2018). These interneurons can be subdivided into different groups based on the expression of neurotransmitters and calcium-binding proteins (Nagayama et al., 2014). In our dataset, we found differential expression of genes related to GABAergic synaptic transmission, including the downregulation of the GABA_A_ receptor and the receptor-associated protein, as well as markers expressed in parvalbumin-positive, vasoactive intestinal peptide (VIP)-positive and dopaminergic interneurons (Fig. 3A). The differential expression of these genes was also validated using qRT-PCR (Fig. 3B). In total, 45 genes were found to be specifically expressed in the neuronal subtypes present in the olfactory bulb (Fig. 3C,D). The majority of these genes were expressed in inhibitory interneurons, and a substantial number of genes was found to be expressed in the cluster related to immature/developing neurons (Tepe et al., 2018).

**Fig. 4.**
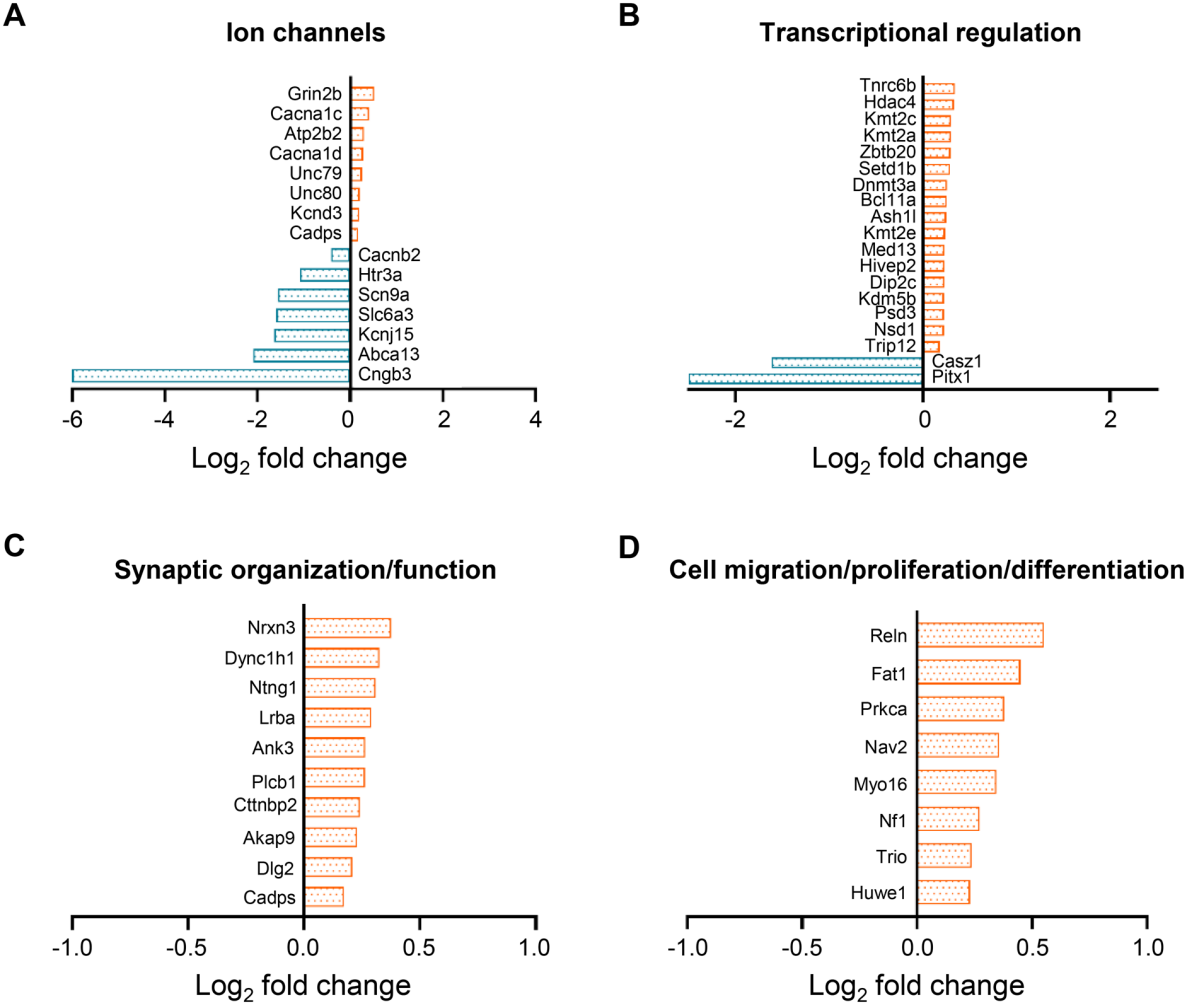
**Identification of ASD-associated genes within the transcriptome analysis.** Comparison of the differentially expressed genes between WT and SEY with the SFARI database resulted in the identification of 56 ASD-associated genes. These can be further subdivided into four categories: (A) Ion channels including several calcium, potassium, and sodium channels; (B) genes involved in transcriptional regulators such as transcription factors but also genes involved in the epigenetic machinery; (C) genes encoding proteins that contribute to synaptic function or organization; (D) genes involved in cell migration, proliferation, and differentiation.

The presence of autism-related genes was evaluated, given the association of Pax6 mutations with ASD and the observation of social behavioral deficits in *Pax6^Sey/+^* mice (Abraham et al., 2010; Kikkawa et al., 2019; Maekawa et al., 2009; Yamamoto et al., 2014). We compared our dataset (FDR<0.05) to the curated database, Simons Foundation Autism Research Initiative (SFARI), containing ASD-associated genes (Arpi and Simpson, 2022; Banerjee-Basu and Packer, 2010). In total, 56 ASD-associated genes were present in our dataset (Fig. 4, Table S1). These could be largely subdivided into four groups based on gene ontology. The first groups comprise several ion channels, including calcium, potassium, and sodium channels (Fig. 4A). Secondly, genes functioning as transcriptional regulators were found, including several genes known to be involved in DNA methylation and chromatin remodeling (Fig. 4B). The remaining two groups contained genes involved in synaptic structure (Fig. 4C) and genes involved in cellular migration/proliferation and differentiation (Fig. 4D). Of these genes, 15 genes were previously identified as direct transcriptional targets of Pax6 (Kikkawa et al., 2019) (Table S1).

## DISCUSSION

In the current study, we conducted a neurobehavioural study on heterozygous *Sey* mice carrying a mutation in the gene coding for the transcription factor *Pax6* (Ochi et al., 2022). We found that *Pax6* mutant mice exhibited impairments in social behavior and olfactory processing. In line with previous studies, our findings provide further evidence for the involvement of Pax6 in the modulation of social performance and support the view that *Pax6* mutant mice could be a relevant model for neurological disorders such as ASD, as previously suggested (Chhabra et al., 2020; Davis et al., 2008; Kikkawa et al., 2019; Kim et al., 2014; Umeda et al., 2010; Yoshizaki et al., 2016), and other conditions characterized by social and sensory processing abnormalities (Arakawa, 2020; Fernández et al., 2018; Magiati et al., 2014; Ricceri et al., 2007; Sledziowska et al., 2020). Moreover, we performed transcriptomic analysis on the olfactory bulb in an attempt to identify molecular mechanisms related to the observed phenotypes in *Pax6* heterozygous mice and its implications in ASD.

We observed that *Pax6^Sey/+^* mice show reduced sociability compared to WT mice, as evidenced by their reduced time spent interacting with stranger mice in the SPSN test. Previous research has also noted that subordinate animals exhibit decreased interest in conspecifics (Kunkel and Wang, 2018). The results of the tube dominance test further supported this observation, as *Pax6^Sey/+^* mice displayed a submissive phenotype characterized by strong withdrawal and more contests lost to WT mice. Previous research into *Pax6* heterozygous rats and their littermates showed aggressiveness and withdrawal behaviors in the open-field test (Yang et al., 2011). We observed no signs of inter-*Pax6^Sey/+^*-WT mice aggression. We only observed withdrawal and subordinate behaviors. In the mouse tube test, conflicts are not resolved by the winner but rather by the loser's withdrawal and retreat (Chou et al., 2021; Harris et al., 2021; Lopez et al., 2024; Pelsőczi et al., 2020). Therefore, we interpret the submissive behavior of *Pax6^Sey/+^* mice as a decreased interest in social approach and increased social avoidance (Defensor et al., 2011).

Given the important role of Pax6 in olfactory bulb development, it is important to exclude that social behavioral deficits are due to the inability to smell (Arbuckle et al., 2015; Dellovade et al., 1998; Nomura et al., 2007; Wesson, 2013). The main olfactory bulb is an essential structure for social recognition since chemically induced anosmia or lesions to the main olfactory bulb impair recognition of conspecifics (Bluthé and Dantzer, 1992; Matochik, 1988; Spehr et al., 2006). Moreover, mice rely on olfactory cues for social interactions, including recognizing conspecifics (Bakker et al., 2022; Dudas et al., 2024; Ryan et al., 2008). Using the olfactory habituation/dishabituation paradigm, we show that *Pax6^Sey/+^* mice are capable of discriminating between non-social and social odors. However, they did not show habituation to the social odor upon repeated presentation. Habituation to social odors is a measure of social recognition memory (Sanchez-Andrade and Kendrick, 2009). This suggests that the social deficits observed in *Pax6^Sey/+^* mice are not due to an inability to detect social odors but rather to impaired processing or recognition of these cues. It has been reported that during olfactory learning, inhibitory control by the GABAergic interneurons on mitral/tufted output neurons increases, thereby controlling the response of these cells to familiar odors (Brennan and Kendrick, 2006; Brennan et al., 1990; Huang et al., 2021; Sanchez-Andrade and Kendrick, 2009). Our transcriptome analysis reveals a significant downregulation of several genes involved in GABAergic neurotransmission, therefore suggesting that the imbalance between excitatory and inhibitory neurotransmission might affect mitral cell output and ultimately result in defective habituation to social odors (Brennan and Kendrick, 2006). Several genes expressed in GABAergic interneurons are of interest because their role has been implicated in social behavior. Of particular interest was the significant downregulation of parvalbumin in *Pax6^Sey/+^* mice. Parvalbumin-positive interneurons are found in the external plexiform layer, where they have dense reciprocal synaptic connections with mitral cells (Kato et al., 2013). In contrast to granule cells, parvalbumin-positive interneurons respond to a broad range of odorants, indicating that they control the mitral cell output across varying levels of sensory input (Kato et al., 2013). It was shown that inhibition of parvalbumin neurons increased the odor-evoked activity of mitral cells without influencing odor selectivity (Kato et al., 2013). Based on these findings, we suggest that a decrease in parvalbumin-expressing interneurons in the olfactory bulb is likely to affect the olfactory function and social behavior in *Pax6^Sey/+^* mice. Parvalbumin-expressing interneurons in other parts of the nervous system have been implicated in social behavior (Deng et al., 2019; Selimbeyoglu et al., 2017; Sun et al., 2020). Reduction of the parvalbumin-expressing neurons in the hippocampus was associated with social memory impairment (Deng et al., 2019), whereas modulating the functionality of parvalbumin-expressing interneurons had a positive effect on social behavior in mice with a disrupted balance between neuronal excitation and inhibition (Selimbeyoglu et al., 2017). Furthermore, post-mortem analysis has revealed a decrease in parvalbumin-expressing interneurons in ASD patients in the cerebral cortex (Hashemi et al., 2017), and studies in ASD mouse models all show a decrease in parvalbumin expression in neurons (Lauber et al., 2018; Shivakumar et al., 2024).

We also observed a significant decrease in VIP in *Pax6^Sey/+^* mice compared to WT littermates. VIP is expressed in GABAergic short-axon interneurons that are found in the external plexiform layer and modulate the activity of the output neurons (Gracia-Llanes et al., 2003; Miller et al., 2014; Nagayama et al., 2014). Besides its important role in odor detection performance, VIP is crucial for maintaining and synchronizing the circadian rhythm in the olfactory bulb (Miller et al., 2014). Previous studies have found that VIP plays an important role in regulating avoidance behavior and social memory storage (Ivanova et al., 2014; Lee et al., 2019; Sun et al., 2020). Wang et al. (2022) identified a group of VIP interneurons that establish direct GABAergic inhibitory connections with mitral cells in the olfactory bulb. These interneurons are essential for odor processing and olfactory discrimination (Wang et al., 2022). Taken together, these cellular and molecular dysfunctions between olfactory sensory neurons, mitral and tufted (M/T) cells and interneurons could manifest as altered olfactory-driven behaviors, including increased submissiveness and impaired social interactions.

In humans, PAX6 heterozygosity is associated with impaired olfaction, with imaging revealing that the olfactory bulb is often underdeveloped even though no differences in its volume were detected using MRI (Mitchell et al., 2003; Sisodiya et al., 2001). Nevertheless, no data is available on the mechanism of functional defects in the olfactory bulb of *PAX6* heterozygous patients. However, based on our results, it seems likely that an imbalance in excitatory and inhibitory neurotransmission contributes to this phenotype. The olfactory bulb is composed of several neuronal subtypes. When analyzing the differentially expressed genes, we found that the majority of these genes are expressed in the periglomerular and granule cells. This is interesting given that Pax6 is mainly expressed in the granule cell and glomerular layer, both layers in which, respectively, granule and periglomerular cells are found (Dellovade et al., 1998). After birth, neurogenesis in the olfactory bulb maintains the generation of interneurons. Pax6 was identified to be important for the generation of dopaminergic cells present in the granule and periglomerular cell layer (Hack et al., 2005; Kohwi et al., 2005). Furthermore, Pax6 has also been detected in the external plexiform layer, where it is involved in the differentiation and maintenance of parvalbumin-positive neurons (Haba et al., 2009). These findings are consistent with our observation that a number of the differentially expressed genes are mainly in granule cells, immature cells, and periglomerular cells.

We also found that several genes were mainly expressed in mitral cells. The role of *Pax6* in mitral cells is limited to the developing olfactory bulb, where *Pax6* is required for the generation of mitral cells from radial glial cells in the ventricular zone (Imamura and Greer, 2013). Downregulation of *Pax6* followed by upregulation of the transcription factors *Tbr1* and *Trb2* are essential for the formation of mitral cells (Imamura and Greer, 2013). Pax6 is also required for proper migration of mitral cells to the olfactory bulb during development (Nomura and Osumi, 2004). It has been suggested that *Tbr1* haploinsufficiency impaired olfactory discrimination and altered the olfactory neuronal circuits in mice (Huang et al., 2019).

Additional evidence supporting the importance of *Pax6* in synaptic function and development is seen in different ASD-associated genes that we identified in our transcriptome analysis. These genes can be subdivided into different groups, including ion channels (e.g. calcium, sodium, and potassium), genes involved in synaptic organization (e.g. NRXN3, ANK3), and genes related to neurogenesis and neuronal migration (e.g. NF1, RELN, CTTNBP2, NTNG1) (Borrie et al., 2021; Duan et al., 2020; Hughes et al., 2022; Liu et al., 2023). The fourth group of ASD-associated genes comprised several transcription factors (e.g. CASZ1, HIVEP2, PITX1, ZBTB20), transcriptional regulators (e.g. MED13, TNRC6B), and genes encoding proteins that are involved in epigenetic regulation. The latter group consists of the DNA methylation enzyme DNMT3A and genes involved in chromatin remodeling of which most of the genes are involved in the modification of histone H3-lysine 4 (H3K4) (e.g. KDM5B, KMT2A, KMT2C, KMT2E) (Brauer et al., 2023; Shen et al., 2014). This suggests that the transcriptional regulation mediated by Pax6 in the olfactory bulb also involves posttranslational modifications of histones. Previously, it was shown that Pax6 induces transcription in the lens by association with methyltransferases (KMT2A, KMT2B) and that decrease in Pax6 results in a different H3K4 methylation status (Kim et al., 2017; Sun et al., 2016). H3K4 methylation is important for normal brain development since mutations associated with either gain or loss of methylation status are associated with neurodevelopmental disorders (Shen et al., 2014). Further research is necessary to evaluate the importance of H3K4 methylation in olfactory bulb development and neurogenesis in adult life as well as investigations regarding the interaction between *Pax6* and these enzymes to control gene expression.

In conclusion, we provide a detailed characterization of the social and olfactory dysfunction in *Pax6^Sey/+^* mice. Plausible mechanisms underlying this defect are related to an imbalance between neuronal excitation and inhibition, more specifically to a decrease in GABAergic neurotransmission. Moreover, we provide evidence for synaptic dysfunction and alterations in epigenetic regulation, two important aspects associated with ASD, in *Pax6^Sey/+^* mice. Given the increased susceptibility for autistic-like features associated with PAX6 polymorphisms, the presence of several important pathological mechanisms similar to ASD and the social behavioral deficits, we propose that further studies of *Pax6^Sey/+^* mice can contribute to our understanding of pathomechanisms of ASD.

## MATERIALS AND METHODS

### Animals

Breeding pairs of *Pax6^Sey/+^* mice (Roberts, 1967) were a generous gift from Professor Veronica van Heyningen's laboratory (MRC Human Genetics Unit, Edinburgh, UK). These mice harbor a mutation in the *Pax6* gene in codon 194, resulting in the replacement of a glycine codon with a nonsense codon and a Pax6 protein truncated before the homeodomain (Hill et al., 1991). These mice were maintained onto the C57BL/6 background. Cohorts of heterozygous Pax6 mutant mice were born and reared at the Mouse Behavior Core Facility mINT at KU Leuven. Male *Pax6^Sey/+^* mice were crossed with female C57BL/6 mice over the same age range. Therefore, we obtained litters that contained both *Pax6^Sey/+^* and WT mice. Mice were separated at weaning (25-28 days of age) and housed with their littermates (3-4 per group) of same-gender and mixed-genotype. Heterozygous Pax6 mutant mice typically exhibit a Sey phenotype. All experiments were performed with male mice aged 20-24 weeks. Animals were housed in standard animal cages under conventional laboratory conditions (12 h/12 h light-dark cycle, 22°C), with *ad libitum* access to food and water. All experiments were conducted during the light phase of their activity cycle. All protocols were reviewed and approved by the animal experiments committee of the University of Leuven (Belgium) and were conducted in accordance with the European Community Council Directive (86/609/EEC).

### Behavioral analysis

#### Social preference and social novelty (SPSN)

The SPSN protocol was used to evaluate social approach as described by Naert et al. (2011). The SPSN is conducted in a three-chambered transparent apparatus with a central chamber (36×28×30 cm) and side chambers (29×28×30 cm) to which access was permitted via sliding doors. The outer chambers contained cylindrical wire cups (11×12 cm) in which stranger mice could be placed. Mice were first habituated to the central chamber for 5 min. After habituation, a same-gender and -age WT stranger mouse was placed on one side under the wire cup (10 min) while an empty wire cup was placed in the opposite side chamber. Stranger mice were selected from parallel breeding groups housed in separate cages in another room to ensure that the two mice never met before the test, and each was used only once per day. Each stranger was pre-habituated to the wire cages, and the location was alternated between the right and left chambers across tested mice. During trials, the test mouse was allowed to explore all three chambers freely for 10 min, and their activity was tracked and recorded using ANY-maze™ Video Tracking System software (Stoelting Co., IL, USA). Time spent sniffing each wire cup was recorded to assess sociability. A total of nine WT and 16 SEY were tested.

#### Tube dominance test

Dominance behavior was assessed in the tube test setup (Benedictus System, Rotterdam, The Netherlands) (van den Berg et al., 2015), comprising a transparent tube (47×2.5 cm) connected to two start boxes (12×8 cm). During the first week, animals were trained individually to cross the tube efficiently (days 1-5, traversing within 30 s) from either start box. During the second week, tournaments were started. The tournaments were conducted in a WT versus *Pax6^Sey/+^* design, and matches were only between animals from different cages that share the same weight and age. Each mouse participated in multiple trials against different opponents of different genotypes to establish a dominance hierarchy. The number of wins was recorded for each mouse. On average, every animal was tested in seven different tournaments. For a match, a mouse was placed in each start box; the outer doors were opened, allowing both mice to enter the tube until the halfway point. When both mice were at the halfway point, the central door was opened, and the match began. The match ended when one mouse forced the other mouse back into its starting box. Following the test, the mice were returned to their original postweaning housing group. In between each training and tournament trial, the setup was cleaned with 70% ethanol. A total of 13 WT and 10 SEY were tested.

#### Odor habituation-dishabituation

This assay evaluates the ability of mice to detect and discriminate different odors (Arbuckle et al., 2015). During the test, mice were placed in a small Type II housing cage with fresh bedding material. After a short acclimatization period (5 min), cotton swabs saturated with odors were presented repeatedly (3×2 min) to the animal. Odors included deionized water, banana (0.1% 2-methylbutyl-acetate in deionized water, Sigma), and grape (0.1% methyl anthranilate in deionized water, Sigma), followed by a social odor. Social odors were presented by moist cotton swaps rubbing through dirty bedding from another uncleaned cage that housed unfamiliar same-sex and -age non-sibling mice. Sniff time was measured using a stopwatch. Habituation to an odor was recorded as a decrease in sniffing/approach time to the cotton swab over the three presentations. Dishabituation was recorded as an increase in sniffing/approach time when presenting a cotton swab with a new odor. A total of 11 WT and 16 SEY were tested.

### RNA-seq

RNA-seq was performed on three biological replicates of WT and four biological replicates of *Pax6^Sey/+^* mice. Each biological replicate contained olfactory bulbs from three mice. Total RNA was isolated from pooled olfactory bulbs derived from WT and *Pax6^Sey/+^* male mice using RNeasy^®^ Lipid Tissue Mini Kit (Qiagen, Germany). RNA sequencing was done by the Genomics Core Leuven (Leuven, Belgium) as a service. Briefly, the Illumina TruSeq Stranded mRNA Sample Preparation Kit was used to prepare sequencing libraries. The Bioanalyzer and the DNA 1000 kit (Agilent Technologies, California, USA) were used to assess library quality and size range. Sequencing was done on Illumina HiSeq4000 with a minimum of 25Mio reads (50 bp, single-end) per sample.

Quality control of raw reads was performed with FastQC v0.11.5. Adapters were filtered with ea-utils v1.2.2.18. Splice-aware alignment was performed with TopHat v2.0.13 against the mouse reference genome Mm10. The number of allowed mismatches was two. Reads that mapped to more than one site of the reference genome were discarded. The minimal score of alignment quality to be included in the count analysis was ten. The resulting SAM and BAM alignment files were handled with Samtools v0.1.19.24. Reads per gene were quantified with HT-Seq count v0.5.3p3. The samples were sequenced and analyzed by Genomics Core Leuven as a service. Count-based differential expression analysis was done with the R-based Bioconductor package DESeq2 (The R Foundation for Statistical Computing, Vienna, Austria) (Love et al., 2014). The Wald test statistic was used to evaluate the differential expression of genes between WT and *Pax6^Sey/+^* mice. Reported *P*-values were adjusted for multiple testing with the Benjamini-Hochberg procedure, which controls the false discovery rate (FDR). A list of differentially expressed genes was selected at an FDR<0.05. Gene ontology was performed using GOseq (Young et al., 2010).

### Statistical analysis

Statistical analyses and graphs were performed using GraphPad Prism, version 9 (La Jolla, CA, USA). The data was first assessed for normal distribution using the Shapiro–Wilk normality test and then subjected to the proper statistical test as indicated in the figure legends and text for each experiment. In SPSN results, normality was violated, and comparisons were made using a non-parametric Wilcoxon matched-pairs signed rank test within-genotype, while comparisons between two independent groups were made with Mann–Whitney *U*-tests. Analysis of tube dominance-subordinate behavior was done using parametric unpaired *t*-tests. Analysis of olfactory exposure: we used repeated measures (RM) ANOVA followed by Sidak's tests, or RM Friedman test ANOVA followed by Dunn's tests, across the three trials of the same odor within-genotype. For dishabituation, we used the paired *t*-test or Wilcoxon matched-pairs signed rank test, as appropriate, between the first trial of an odor and the last exposure to a previous odor. Analysis of qPCR results was done using unpaired *t*-tests for pairwise comparisons. We assumed 0.05 is a level of significance. Results are expressed as mean±s.e.m.

## Supplementary Material

10.1242/biolopen.061647_sup1 Supplementary information

File S1. This file list all differentially expressed genes that were identified in the RNAseq analysis.
