# Supplementary material for: Gene expression differences in the olfactory bulb associated with differential social interactions and olfactory deficits in Pax6 heterozygous mice
Source: Biol Open. 2025 Feb 4;14(2):BIO061647. doi: 10.1242/bio.061647 (PMC11832127; doi:10.1242/bio.061647)
Supplement: Supplementary information [file biolopen-14-061647-s1.pdf]

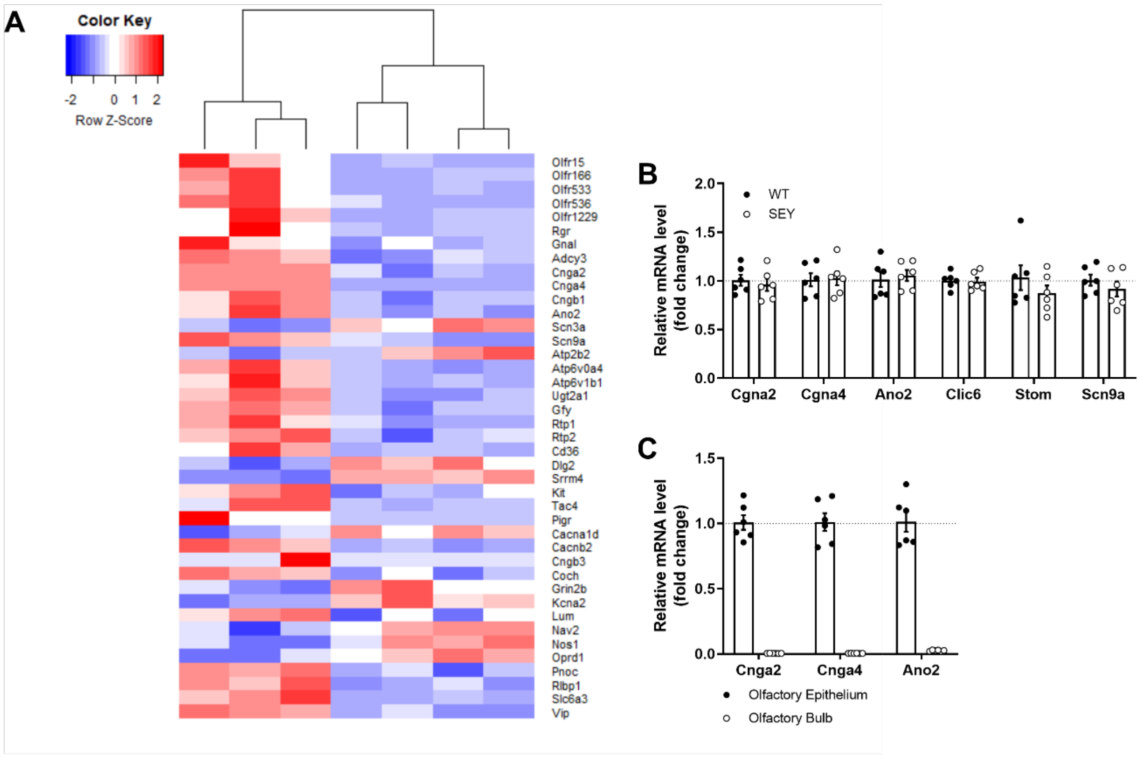

Fig. S1. Differentially expressed genes between WT and *Pax6*$^{Sey/+}$ (SEY) mice in the olfactory epithelium **A.** Heatmap showing the differential expression of genes related to the olfactory epithelium, (WT: n=3, SEY: n=4). **B.** The differential expression could not be validated using a selected group of markers using qRT-PCR. The tissue used for this analysis was carefully dissected in order to avoid cross-contamination, (WT: n=6, SEY: n=6); **C.** Confirmation that the genes are specifically expressed in the olfactory epithelium and that no contamination occurred in the newly dissected tissues (WT: n=6, SEY: n=6).

Biology Open • Supplementary information

Table S1. Autism spectrum disorder-associated genes identified in olfactory bulb transcriptome analysis

Gene	Full name	GO Function (Reference GO:ensembl)
**Transport**		
Abca13	ATP binding cassette subfamily A member 13	Lipid transport
Atp2b2	ATPase, Ca++ transporting, plasma membrane 2	Ca^{2+} transport; intracellular Ca^{2+} homeostasis
Cacna1c	calcium channel, voltage-dependent, L type, alpha 1C subunit	Ca^{2+} channel
Cacna1d*	calcium channel, voltage-dependent, L type, alpha 1D	Ca^{2+} channel
Cacnb2	Calcium channel, voltage-dependent, beta 2 subunit	Ca^{2+} channel
Cadps*	calcium dependent secretion activator	protein involved in Ca^{2+} regulated exocytosis of secretory vesicles/ synapse vesicles
Cngb3	cyclic nucleotide gated channel beta 3	transport
Grin2b	glutamate receptor, inotropic, N-methyl D-aspartate 2B	NMDA receptor (calcium permeability)
Htr3a*	5-hydroxytryptamine (serotonin) receptor 3A	ion channel
Kcnd3	potassium voltage-gated channel subfamily D member 3	K channel
Kcnj15	potassium voltage-gated channel subfamily J member 15	inward rectifying K-channel
Scn9a	sodium voltage-gated channel alpha subunit 9	Na^{+} channel
Slc6a3	Solute carrier family 6 (neurotransmitter transporter), member 3	DAT solute carrier - terminates the action of dopamine
Unc79	unc-79 homolog, NALCN channel complex subunit	component of sodium channel complex
Unc80	unc-80 homolog, NALCN activator	component of sodium channel (NALCN) complex
**Synapse function and organization**		
Akap9	A kinase (PRKA) anchor protein 9	Binding type II regulatory subunits of protein kinase A; suggested role in organization of post-synapse
Ank3*	ankyrin 3	Synapse organization
Cttnbp2	cortactin binding protein 2	cytoskeleton organization; synapse organization; dendritic spine morphogenesis
Dlg2	discs large MAGUK scaffold protein 2	Encodes protein that may interact with post-synaptic sites; receptor clustering postsynaptic
Dync1h1	dynein cytoplasmic 1 heavy chain 1	dynein chain; molecular motors; axonal retrograde transport
Lrba	LPS-responsive vesicle trafficking, beach and anchor containing	Vesicular trafficking
Nrxn3*	neurexin 3	Synapse assembly; vesicle exocytosis
Ntng1*	netrin G1	Axon guidance during development
Plcb1*	phospholipase C, beta 1 (phosphoinositide-specific)	Phospho-inositol catabolic process (produces diacylglyerol; postsynaptic transmission
Cadps	calcium dependent secretion activator	protein involved in Ca^{2+} regulated exocytosis of secretory vesicles; synapse vesicles
**Transcriptional regulators**		
Ash1l	Ash1 (absent, small, or homeotic)-like (Drosophila)	histone-lysine-N-methyltransferase; encodes transcriptional activators; regulation of gene expression
Bcl11a*	B-cell CLL/lymphoma 11A (zinc finger protein)	Encodes zinc finger; transcriptional regulation; BAF complex, chromatin remodeling
Casz1	castor zinc finger 1	Zinc finger transcription factor; gene transcriptional regulation by histone interaction
Dip2c	disco interacting protein 2 homolog C	Transcription factor in CZS

Biology Open • Supplementary information

Hdac4	histone deacetylase 4	Transcription regulator
Hivep2	HIVEP zinc finger 2	Encodes zinc finger containing transcription factors
Med13	mediator complex subunit 13	Encodes coactivator involved in regulated transcription of all RNA polymerase II dependent genes
Nsd1	nuclear receptor binding SET domain protein 1	Enhances Androgen receptor activation, transcriptional regulation , histone methyltransferase
Pitx1*	paired-like homeodomain 1	Transcription factor
Psd3	pleckstrin and Sec7 domain containing 3	guanine nucleotide exchange factor; regulation of gene transcription
Tnrc6b*	Trinucleotide repeat containing 6B	RNAi-mediated gene silencing required for miRNA-dependent translational repression
Trip12	Thyroid hormone receptor interactor 12	E3 ubiquitin protein ligase plays a role in DNA damage response (cancer)
Zbtb20	Zinc finger and BTB domain containing 20	transcription factor
Dnmt3a*	DNA (cytosine-5-)-methyltransferase 3 alpha	required for DNA methylation during development (maternal and paternal imprinting)
Kdm5b	Lysine (K)-specific demethylase 5B	histone demethylase
Kmt2a	Lysine (K)-specific methyltransferase 2A	Transcriptional regulator in early development; methyltransferase on histones
Kmt2c	Lysine (K)-specific methyltransferase 2C	histone methylation
Kmt2e	Lysine (K)-specific methyltransferase 2E	histone methylation
Setd1b	SET domain containing 1B	histone methyltransferase
**Cell migration/ proliferation /growth**		
Huwe1	HECT, UBA and WWE domain containing 1, E3 ubiquitin protein ligase	X-chromosome linked; protein ubiquitination; regulates differentiation and proliferation of MYNC
Nav2*	neuron navigator 2	Cellular growth and migration
Nf1*	neurofibromin 1	Negative regulator of cell proliferation
Prkca	protein kinase C alpha	DAG-dependent protein kinase - cell proliferation, differentiation
Myo16	myosin XVI	Motor protein, actin binding - neuron projection - development
Trio	Trio Rho guanine nucleotide exchange factor	exchange GDP by GTP - cell-matrix and cytoskeletal rearrangements necessary for cell migration and cell growth
Reln	Reelin	ECM protein important for cell positioning and neuronal migration during brain development
Fat1	FAT atypical cadherin 1	Controlling cell proliferation during development
**Others**		
Lrp1	LDL receptor related protein 1	receptor involved in intracellular signaling, lipid homeostasis and clearance of apoptotic cells
Ubn2	ubinuclein 2	unknown
Wdfy3*	WD repeat and FYVE domain containing 3	phosphatidylinositol binding protein; autophagy

* indicates PAX6 regulated genes identified by Kikkawa *et al.* 2019

File S1. This file list all differentially expressed genes that were identified in the RNAseq analysis.

Available for download at
https://journals.biologists.com/bio/article-lookup/doi/10.1242/bio.061647#supplementary-data

Biology Open • Supplementary information
